# Race and nativity are major determinants of tuberculosis in the U.S.: evidence of health disparities in tuberculosis incidence in Michigan, 2004–2012

**DOI:** 10.1186/s12889-017-4461-y

**Published:** 2017-06-02

**Authors:** Grace A. Noppert, Mark L. Wilson, Philippa Clarke, Wen Ye, Peter Davidson, Zhenhua Yang

**Affiliations:** 1 Department of Epidemiology, University of Michigan School of Public Health, Ann Arbor, MI USA; 2 Center for the Study of Aging and Human Development, Duke University, Durham, NC USA; 3 Survey Research Center, Institute for Social Research, University of Michigan, Ann Arbor, MI USA; 4 Department of Biostatistics, University of Michigan School of Public Health, Ann Arbor, MI USA; 5 Michigan Department of Health and Human Services, Lansing, MI USA

**Keywords:** Tuberculosis, Health disparities, Social determinants of health, Infectious disease

## Abstract

**Background:**

The incidence of TB in Michigan was 1.5 per 100,000 people in 2012, roughly half the U.S. incidence. Despite successes in TB control, disparities in TB still exist in Michigan, particularly by race, age, and nativity. A major challenge in understanding disparities in TB burden is distinguishing between TB cases resulting from recent transmission and those resulting from reactivation of latent TB infection, information critical to tailoring control strategies. We examined nine-year trends in tuberculosis (TB) incidence patterns for the entire population of Michigan, and within demographic subgroups.

**Methods:**

Using a cross-sectional study of TB surveillance data, we analyzed 1254 TB cases reported in Michigan during 2004–2012. Cases included were those for whom both spoligotyping and 12-locus-MIRU-VNTR results were available. Using a combination of the genotyping information and time of diagnosis, we then classified cases as resulting from either recent transmission or reactivation of latent TB infection. We used multivariable negative binomial regression models to study trends in the TB incidence rate for the entire population and by race, nativity, gender, and age.

**Results:**

Overall, the incidence rate of TB declined by an average of 8% per year—11% among recently transmitted cases, and 9% among reactivation cases. For recently transmitted disease, Blacks had an average incidence rate 25 times greater than Whites, after controlling for nativity, gender, and age. For disease resulting from latent TB infection Asians had an average incidence rate 24 times greater than Whites, after controlling for nativity, gender, and age.

**Conclusions:**

Disparities in incidence persist despite ongoing TB control efforts. Greater disparities were observed by race and nativity demonstrating some of the ways that TB incidence is socially patterned. Reducing these disparities will require a multi-faceted approach encompassing the social and environmental contexts of high-risk populations.

**Electronic supplementary material:**

The online version of this article (doi:10.1186/s12889-017-4461-y) contains supplementary material, which is available to authorized users.

## Background

The social underpinnings of tuberculosis (TB) disease have long been documented both in historical narratives and scientific literature. Yet, disparities in the incidence of TB related to nativity (where a person is born), race, and socio-economic status (SES) continue to persist despite organized TB control efforts. Applying a social determinants of health framework to infectious etiologies, specifically TB, could shed light on more distal social and environmental factors that may be inhibiting our ability to reduce enduring disparities in TB [1]. Such an approach may be able to shift our understanding from delineation of risk factors to a more comprehensive understanding of the processes producing such risk factors [2].

Despite a resurgence of TB in the U.S. from 1986 to1992, incidence is now at its lowest (3.2 per 100,000 in 2012) since routine reporting began in 1953 [3, 4]. That decline, however, has recently stagnated [3, 5], in both urban and rural populations [6, 7] and among foreign-born persons [5]. In addition, incidence of TB in the U.S. is much higher among racial/ethnic minorities, people of lower SES, those with HIV, the homeless, and the incarcerated [5, 8–11]. Consistent with various studies that have examined the effects of SES on health [12], TB incidence shows an SES gradient, whereby people of lower SES experience greater risk of TB—a gradient much steeper among U.S.-born cases [10, 13, 14].

Many studies have reported disparities in TB incidence in the U.S., particularly racial disparities [15–19]. However, few studies have contextualized these disparities in the larger framework of social and environmental determinants of health. In the late 1980s and early 1990s, several studies examined the social and geographic context of TB cases as means to understand the increase in TB incidence following the HIV epidemic [14, 20, 21]. More recently, studies in Washington State examined the relationship between neighborhood socioeconomic disadvantage and TB incidence and disease progression finding that residence in an area with greater neighborhood disadvantage is associated with increased TB incidence, accelerated progression of disease, and genotypic clustering, particularly among U.S.-born cases [22–24]. Recent studies seeking to understand the drivers of these persistent disparities posit that SES and/or an unequal burden of TB risk factors may be confounding our understanding of racial and ethnic disparities in TB in the U.S. [15, 16]. It may be that investigations of the social and geographic context of individuals hold clues for understanding the drivers of TB disparities in different contexts, and for designing context-specific interventions to ameliorate them.

In the state of Michigan TB control has largely been successful; the incidence rate is consistently lower than the national average [15]. Yet, despite Michigan being a low-burden state, TB remains a notable public health issue, particularly among socially vulnerable populations. At present about 75% of TB cases occur in the Detroit Metro Area while only 39% of the Michigan population resides there [15, 25]. Moreover, about half of Michigan TB cases are foreign-born, compared to 63% of the national TB cases in 2012 [4]. In 2010, less than 8% of the Michigan population was foreign-born, compared to 13% of the U.S. population [26]. Understanding the differential impacts of social factors on TB infection and disease, and specifically how such factors differ for recently transmitted versus cases resulting from reactivation of latent TB infection (LTBI), will help reduce risk and improve treatment.

Much of the investigation of TB in the U.S. in recent decades has focused on high-burden settings. However, as we move from strategies aimed at controlling TB to strategies aimed at eliminating TB, it is increasingly important to understand the patterns of TB incidence in low-burden settings. This is critical given the levelling off of TB incidence in the U.S. in recent years [27]. Recent reports have emphasized the importance of local approaches to TB control; approaches that take into account context (including socio-demographic context) when thinking about how to best design TB control interventions [28, 29]. Having a more thorough understanding of TB epidemiology in low burden settings, and particularly understanding the local context can allow the development of more effective TB control strategies to achieve TB elimination.

Using Michigan surveillance data from 2004 to 2012, we examined TB incidence patterns for the entire population of Michigan, and within population subgroups. We used both genotypic and temporal data to investigate trends separately for cases due to recent transmission and those due to reactivation of LTBI. We specifically sought to document disparities in TB incidence by race, nativity, gender, and age in Michigan with the aim of laying the foundation for future studies that can explore the mechanisms underlying such disparities.

## Methods

### Study population and data collection

**Fig. 1:**
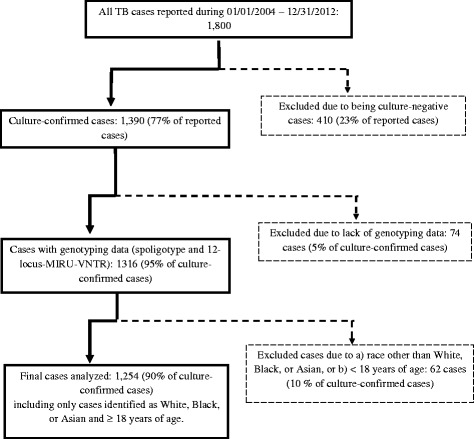
Flowchart illustrating the selection of the study sample from the 1800 total TB cases reported in Michigan during 2004 to 2012

A total of 1800 TB cases were reported to the Michigan Department of Health and Human Services during January 1, 2004 - December 31, 2012 (Fig. 1). The gold standard of TB diagnosis is whether TB culture grows from inoculation with a TB sputum and/or tissue sample in a laboratory setting. The resulting cultures allow for genotyping of TB isolates giving the ability to infer genotypic clusters via two genotypic measures: spoligotyping and 12-locus-MIRU-VNTR. Therefore, we limited our analyses to only those cases confirmed with a positive culture. Of the 1800 total cases, 1390 (77%) cases were culture-confirmed; 410 (23%) cases were those without microbiological confirmation, or culture-negative.

We used the first isolate from each case and only a single isolate was used per case. As with previous studies, a genotypic TB cluster was defined as two or more cases with identical spoligotype and 12-locus MIRU genotyping patterns in addition to a diagnostic date within a one year time period of one another [7, 30]. Such clusters are not necessarily spatial clusters, and could occur over more than one year if the cases were connected by another case with an identical genotypic pattern within the one-year time frame [7]. If a case did not meet this definition, it was classified as a non-clustered case [7]. With this time-restricted genotypic cluster definition, clustered cases were considered as a proxy for cases resultant from recent transmission; non-clustered cases to be a proxy for cases resultant from reactivation of LTBI. The inclusion of a time requirement allows for greater specificity in the classification of a genotypic cluster as a case resulting from recent transmission. Thus, cases were excluded if they did not have both a spoligotype and a 12-locus-MIRU-VNTR result. Of the 1390 culture-confirmed cases, 1316 (95% of all culture-confirmed cases) had both genotypic measures.

Demographic and clinical characteristics of the sample were drawn from de-identified TB surveillance data collected by the Michigan Department of Health and Human Services using the “Report of a Verified Case of TB” form [31]. Classifications of race, nativity, gender, and age were based on demographic data collected from the above form. We only included participants who self-identified as Black, Asian, or White in our racial classification. We focused only on race rather than race/ethnicity because the data did not capture multiple ethnic categories. Further, only 10% of the sample identified as Hispanic ethnicity. In order to ensure comparability with previous studies, age was defined as 18–64 or ≥65 years old [7, 30]. Cases under 18 years of age were excluded due to the difficulty in accurately ascertaining pediatric cases [32]. Gender and nativity were dichotomized as male or female and U.S.-born or foreign-born, respectively. The study sample represents 70% of the total number of reported TB cases in Michigan during the study period.

The study was approved by the Health Sciences and Behavioral Sciences Institutional Review Board at the University of Michigan.

### Statistical analyses

Using a cross-sectional study of TB surveillance data, we analyzed 1254 TB cases reported in Michigan during 2004–2012. Incidence rates for the study time period were calculated overall for the study population, clustered and non-clustered cases separately, and then by race, age, gender and nativity. To generate the denominators for the total population and subgroup incidence rate calculations, population-level characteristics for Michigan were obtained from the American Community Survey through the U.S. Census Bureau [33]. Enumerating the population by nativity, race, gender, and age is considered identifiable data according to the U.S. Census. Thus, in order to obtain these subgroup population estimates, we took the available population numbers (for example, the number of persons who are foreign-born, white, and male) and applied the age proportions reported in the American Community Survey.

We first visually examined the trends in incidence both overall and among subgroups of race, nativity, gender, and age. We plotted the incidence rates over time and calculated the confidence intervals based on the Poisson distribution following the method developed by Buchanan [34] in Microsoft Excel (2011).

Negative binomial regression models with a log link were then developed to examine temporal changes in incidence overall and among clustered and non-clustered cases, and by subgroups of race, nativity, gender, and age. We used negative binomial models because they are appropriate for count data without relying on the assumption of a mean equal to the variance. The distribution of our data was such that the variance was substantially greater than the mean. We first modeled the effect of each demographic factor by time (year) separately. There was no significant effect of time, which therefore was removed from the model. We then used a multivariable negative binomial regression model with a log link to analyze the average incidence rate ratio by subgroups of the population, including all demographic factors. In our models, we assumed that each case contributed one person-year of follow-up time.

All regression analyses were conducted in SAS V9.4 and statistical significance was assessed with a two-tailed alpha level of 0.05.

## Results

Of the 1254 cases in the sample, 473 (38%) fit our criteria for a clustered case, while 781 (62%) were considered non-clustered cases. The 473 clustered cases belonged to 95 unique clusters with the size ranging from 2 to 51 cases.

### Characteristics of the study population

Of the 1254 cases analyzed in this study 44% were foreign-born and 55% U.S.-born (Table 1). The sample was 33% White, 42% Black, and 25% Asian. Cases ranged from 18 years of age to 104 years of age with a median of 49 years of age. 60% of the cases were male, 40% female (Table 1). Finally, 70% of the cases had pulmonary TB, 22% extrapulmonary TB, and 8% had both pulmonary and extrapulmonary TB (Table 1). The socio-demographic composition of the study sample was similar to that of all TB cases diagnosed in Michigan during the study period (Additional file 1: Table S1).

**Table 1:** Comparison of the distribution of selected demographic and clinical characteristics among those cases included in the study sample to those cases excluded from the study sample

Risk factor	Study sample (*n* = 1254)		Excluded from study sample (*n* = 546)		*P*-Value
	N	%	N	%	
Race					
White	419	33.4	199	36.5	<0.0001
Black/African American	525	41.9	196	35.9	
Asian	310	24.7	118	21.6	
American Indian/Alaskan Native			5	0.92	
Native Hawaiian or Other			14	2.6	
Unknown			11	2.0	
Missing			3	0.55	
Age groups (years)					
<18			124	22.7	<0.0001
18–64	939	74.9	324	59.3	
65+	315	25.1	98	18.0	
Gender					
Male	750	59.8	297	54.4	0.04
Female	504	40.2	248	45.4	
Missing			1	0.18	
Nativity					
Foreign-born	557	44.4	241	44.1	0.58
US-born	694	55.3	302	55.3	
Missing	3	0.24	3	0.55	
Site of disease					
Pulmonary	880	70.3	308	56.4	<0.0001
Extrapulmonary	275	22.0	188	34.4	
Both	97	7.8	46	8.4	
Missing	2		4	0.73	

Classifications of race, age, gender, nativity, and site of TB disease were defined based on the Report of Verified Case of TB form developed by the Centers for Disease Control and Prevention

*P*-values are based on the chi-squared test

### State-wide incidence rate trends

From 2004 through 2012, the overall incidence rate of TB declined from 2.69 cases per 100,000 persons in 2004 to 1.28 cases per 100,000 in 2012. The average annual percent decline was 8.0%, (95% CI [−0.49, 15.7], *P* = 0.06).

The incidence rate for both clustered and non-clustered TB declined over the time period. The decline in the incidence rate was the largest among clustered TB cases falling from 1.01 per 100,000 in 2004 to 0.36 per 100,000 in 2012. This corresponds to an average annual decline of 11.2% (95% CI [−0.60, 21.6), *P* = 0.06). The incidence rate of non-clustered TB was 1.68 per 100,000 persons in 2004 and 0.92 per 100,000 persons in 2012, corresponding to an average annual decline of 8.5% (95% CI [−0.06, 16.4], *P* = 0.05). The difference between the average annual decline in the incidence rate for clustered and non-clustered TB does not appear to be significant given the overlapping 95% confidence intervals.

Over the nine-year study period, the proportion of cases classified as clustered in the study sample decreased while the proportion of non-clustered cases increased. In 2004, clustered cases accounted for 38% of the study sample versus 28% in 2012.

### Subpopulation incidence rate trends

**Fig. 2:**
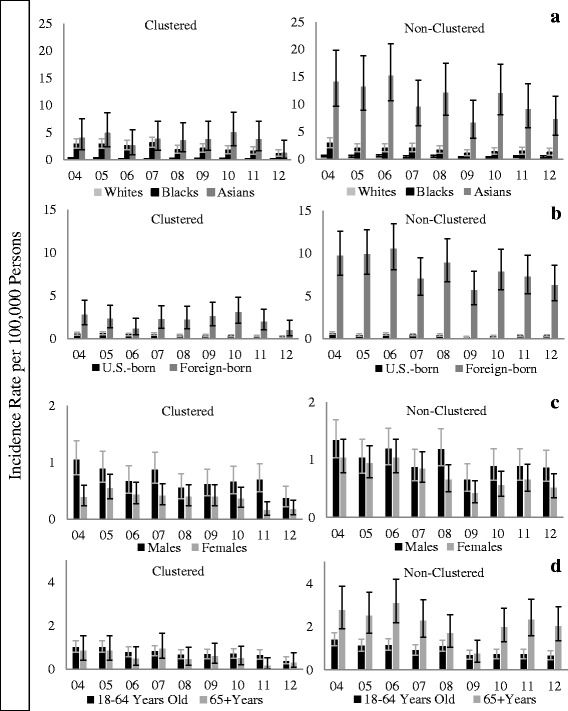
The incidence rate of clustered and non-clustered TB cases by race, nativity, gender, and age. For all figures, the error bars correspond to the 95% confidence interval. **a**. Comparison by race. Light grey bar represents the incidence rate per 100,000 persons for Whites. Black bar represents the incidence rate per 100,000 persons for Blacks. Medium grey bar represents the incidence rate per 100,000 persons for Asians. **b**. Comparison by nativity. Black bar represents the incidence rate per 100,000 persons for U.S.-born persons. Grey bar represents the incidence rate per 100,000 persons for Foreign-born persons. **c**. Comparison by gender. Dark grey bar represents the incidence rate per 100,000 persons for males. Light grey bar represents the incidence rate per 100,000 persons for females. **d**. Comparison by age Dark grey bar represents the incidence rate per 100,000 persons for those 18–64 years old. Light grey bar represents the incidence rate per 100,000 persons for those 65 years of age and older

No significant differences were found in the rate of decline in the incidence comparing Blacks and Asians to Whites (Fig. 2a). Overall, Blacks had an average annual decline in the incidence rate of 9.0% (95% CI [−3.5, 20.0]), Asians 4.1% (95% CI [−9.2, 15.7]) and Whites 11.0% (95% CI [−0.7, 21.4]) (*P* = 0.80 and *P* = 0.41 for Blacks and Asians compared to Whites, respectively). Among the clustered cases, Blacks had an average annual decline in the incidence rate of 10.4% (95% CI [−5.4, 23.8]), Asians 7.5% (95% CI [−12.1, 23.6]) and Whites 14.8% (95% CI [−1.7, 28.6]) (*P* = 0.68 and *P* = 0.54 for Blacks and Asians compared to Whites, respectively) (Fig. 2a). Among non-clustered cases, Blacks had an average annual decline in the incidence rate of 11.1% (95% CI [−2.9, 23.2]), Asians 5.3% (95% CI [−7.9, 16.8]), and Whites 10.4% (95% CI [−2.0, 21.4]) (*P* = 0.94, *P* = 0.55 for Blacks and Asians compared to Whites, respectively) (Fig. 2a).

Similarly, there were not significant differences in the decline rate by nativity (Fig. 2b). Overall, the U.S.-born had an average annual decline of 9.3% (95% CI [−3.0, 20.2]) and foreign-born 7.2% (95% CI [−3.7, 17.0]) (U.S.-born vs. foreign-born: *P* = 0.79). Among clustered cases, the U.S.-born had an average annual decline in the incidence rate of 15.3% (95% CI [−0.5, 28.4]) and foreign-born 9.5% (95% CI [−5.0, 21.9]) (U.S.-born vs. foreign-born: *P* = 0.56). Among non-clustered cases, the U.S.-born had an average annual decline in the incidence rate of 11.2% (95% CI [−1.2, 22.1]) and foreign-born 7.0% (95% CI [−4.4, 17.2]) (U.S.-born vs. foreign-born: *P* = 0.60).

There were no differences in the rate of decline in the incidence by gender (Fig. 2c).

Overall, males had an average annual decline in the incidence rate of 3.3% (95% CI [−8.8, 14.0 and females 15.3% (95% CI [3.4, 25.8]) (male vs. female: *P* = 0.14). Among clustered cases, males had an average annual decline of 9.4% (95% CI [−6.6, 23.1]) and females 14.5% (95% CI [−4.1, 29.7]) (male vs. female: *P* = 0.66). For non-clustered cases, males had an average annual decline in the incidence rate of 2.2% (95% CI [−10.4, 13.4]) and females 17.6% (95% CI [−6.4, 27.8]) (male vs. female: *P* = 0.06). While the interaction between time and gender among non-clustered cases approaches significance with a *p*-value of 0.06, it was not significant with a cut point of 0.05.

Likewise, the two age groups did not differ in the rate of decline in the incidence (Fig. 2d). Overall, the 18–64 year age group had an average annual decline in the incidence rate of 11.4% (95% CI [0.41, 21.2]) and the 65+ age group 4.4% (95% CI [−8.7, 16.0]) (65+ vs. 18–64: *P* = 0.39). Among clustered cases, the 18–64 year age group had an average annual decline in the incidence rate of 10.6% (95% CI [−4.6, 23.5]) per year and the 65+ age group 13.9% (95% CI [−7.8, 31.2]) (65+ vs. 18–64: *P* = 0.79). This trend was reversed among the non-clustered cases, the 18–64 age group had an average annual decline in the incidence rate of 14.3% (95% CI [4.0, 23.5]) compared to the 65 + age group of 3.9% (95% CI [−9.3, 15.4] (65+ vs. 18–64: *P* = 0.19).

### Comparison of average incidence rate among subpopulations

We next evaluated whether there were significant differences in the average incidence rate ratio across subgroups for both clustered and non-clustered TB cases. Given no significant differences in the decline rate were found among subgroups when the demographic factors were examined one at a time, interactions between time and demographic factors were not included in the multivariable analysis. The greatest disparities in the average incidence rate were observed by race and nativity among the clustered cases (Table 2). However, there were also stark disparities evident among the non-clustered cases.

**Table 2:** Incidence rate ratio of TB according to selected socio-demographic characteristics in Michigan, 2004–2012

Variable	Clustered cases		Non-clustered cases	
	N	IRR (95% CI)	N	IRR (95% CI)
	479		775	
Race				
White	107	Ref.	312	Ref.
Black	296	24.6 (15.5, 38.8)	229	7.7 (5.3, 11.1)
Asian	76	18.6 (10.5, 32.9)	234	23.5 (16.3, 35.8)
Nativity				
U.S.-born	359	Ref.	335	Ref.
Foreign-born	117	10.0 (6.6, 15.2)	440	7.0 (5.0, 9.8)
3 missing				
Gender				
Male	313	Ref.	437	Ref.
Female	166	0.61 (0.41, 0.91)	338	0.74 (0.54, 1.0)
Age				
18–64 Years	414	Ref.	525	Ref.
65+ Years	65	0.83 (0.54, 1.3)	250	2.6 (1.9, 3.5)

Classifications of race, age, gender, nativity, and site of TB disease were defined based on Report of Verified Case of TB form developed by the Centers for Disease Control and Prevention

Models based on multivariable negative binomial regression models

Clustered and non-clustered cases were modeled separately with all four demographic characteristics included in models: race, nativity, gender, and age

*IRR* incidence rate ratio

*Ref*. reference group

Among clustered TB cases, Blacks had an average incidence rate 25 times greater than Whites, with Asians at 19 times greater incidence than Whites, after controlling for nativity, gender, and age (Table 2). In the same model, the foreign-born had an average incidence rate 10 times greater than the U.S.-born after controlling for race, gender, and age (Table 2).

Disparities were also observed in the non-clustered model, particularly by nativity. In the non-clustered model, the foreign-born had an average incidence rate 7 times greater than that of the U.S.-born when controlling for race, gender, and age (Table 2). Racial disparities were also observed: Blacks had an average incidence rate 7 times greater than that of Whites, Asians 24 times greater than that of Whites, when controlling for nativity, gender, and age (Table 2).

In addition, further investigation of the effects of race and nativity showed that nativity is a confounder of the relationship between race and TB incidence. In the clustered model, we saw that racial disparities were reduced when nativity was included in the model for both Blacks and Asians. In the non-clustered model, the racial disparities were slightly attenuated for Blacks when nativity was included in the model. However, racial disparities were amplified for Asians when accounting for nativity (results shown in Additional file 2: Table S2).

## Discussion

This study analyzed temporal changes in TB incidence patterns in Michigan during 2004–2012, with particular attention to subgroups by race, nativity, gender, and age. We found the incidence rate of TB to be declining overall in Michigan both for recently transmitted cases and reactivated cases. Our results suggest both ongoing transmission and reactivation of LTBI are actively contributing to the burden of TB disease in Michigan. Notably, we found significant subgroup disparities in the average incidence rate, particularly by race and nativity.

On the whole TB incidence is declining in the U.S. We observed an average yearly percentage decline of 8% in the incidence, greater than the average percentage decline (5%) reported for the U.S. as a whole in this time period [4, 35–42]. The greater decline in Michigan might be partially explained by a population composition that differs from many of the more populous states contributing to the national TB burden such as California, Texas, New York, and Florida who accounted for nearly half of all TB cases in 2014 [43]. The higher TB incidence rate in these states may be driven by a larger proportion of foreign-born populations in these state as compared with Michigan [44].

Our findings show that both ongoing transmission and reactivation of LTBI are contributing to TB incidence in Michigan. Based on our analysis, an average of 62% of the cases in Michigan resulted from reactivation of LTBI. Studies in Arkansas have reported similar findings, though with smaller proportions of cases attributable to reactivation [7, 30]. Based on our analysis, TB control efforts need to implement additional measures that can both reduce *Mycobacterium tuberculosis* (MTB) transmission and reduce reactivation of LTBI. Routine contact tracing is a key component of the TB control strategy in Michigan; however, additional routine screening for LTBI and active TB, followed by timely appropriate treatment may be beneficial among certain sub-populations in Michigan.

Although the concept of race is widely recognized as a social construction, it represents a strong predictor of health and disease in the U.S. Racial classifications in the U.S. are based on a social definition of race, rather than a biological or genetic definitions [45]. Racial differences in the U.S. reflect both historic and contemporary differences in power, status, and access to resources [46]. Accordingly, differences by race, and the meaning of those differences, must be assessed when examining health disparities in the U.S. context.

In general, we did not find significant differences in the TB decline rate in our subgroup analyses. However, there was evidence of disparities in the average incidence rate, particularly by race and nativity. Among recently transmitted cases, the highest risk racial group was Blacks while among reactivation of LTBI cases it was Asians. The evidence is mixed regarding the racial distribution of TB. While some studies have also reported the greatest racial disparity in TB incidence between Blacks and Whites [47], the national surveillance data suggests the greatest racial disparities exist between Asians and Whites [43]. Our multivariable models allowed us to explore these disparities while accounting for the effects of nativity. For example, among both recently transmitted cases and reactivation of LTBI cases, we demonstrated that nativity confounded the relationship between race and TB disparities. In some cases, the racial disparities were reduced after accounting for nativity, while in other cases the disparities were amplified after accounting for nativity (results shown in Additional file 2: Table S2). Thus, accounting for nativity is critical to understanding the relationship between race and TB incidence.

Disparities by nativity were evident among both cases resulting from recent transmission as well as those resulting from reactivation of LTBI. Immigrants may be infected in their country of origin and may reactivate sometime later when in the U.S. [48–50]. However, the disparity observed between foreign-born and U.S.-born persons among the recently transmitted cases suggests they are also at risk for acquiring TB infection while in the U.S. This may be resultant from increased exposure to active TB cases among other recent immigrants or it could it be a reflection of their social and environmental circumstances once in the U.S.

Our study is one of only a few to use a multivariable approach to disentangle the differential impact of socio-demographic factors on disparities in TB incidence in the U.S. Bivariate analyses of the socio-demographic patterning of TB risk miss important relationships that are only uncovered when controlling for other factors. Other studies that have used a multivariable approach have also found that social vulnerability based on minority race/ethnicity, nativity, and income was a better predictor of TB incidence than traditional TB risk factors such as over-crowding living conditions or high population density [51].

During the 19th and early 20th centuries, TB was thought to be endemic in the U.S., particularly among those living in poor, crowded housing without access to basic resources: clean water, sanitation, ample food [52, 53]. Yet, in recent decades the focus in infectious disease control in the U.S. has shifted to individual-level predictors of risk and disease, often ignoring the social and environmental context of individuals that may be a more salient predictor of risk. While we recognize that the same types and magnitudes of resource deprivation that occurred more than a century ago are not the same today, our findings indicate that, despite organized TB control efforts, contemporary disparities are still important in explaining TB patterns today, suggesting that it could be important to again address the social and environmental context in which TB cases are arising. It is not sufficient to simply describe the patterns of TB incidence without regard to how these factors are working together to augment risk in certain populations.

Additionally, despite our knowledge of the highly social nature of TB, control efforts in the U.S. rarely emphasize the social and environmental context as means for intervention. Recently, the World Health Organization has begun to recognize the inextricable links between social and economic determinants of TB incidence globally [52, 54]. Globally, the burden of TB often disproportionately falls on the most vulnerable populations. Hargreaves et al. suggests that global TB control needs to develop interventions that simultaneously incorporate the traditional biomedical approaches as well as approaches that address the social determinants of health [54]. These efforts have mainly been targeted to high-burden TB countries in order to reduce the global incidence of TB. However, we advocate for an extension of this expansive view of TB control to low burden TB settings as TB disparities often persist in these settings. While many studies show social factors ranging from neighborhoods to social cohesion to be meaningful predictors of TB risk, we now need to move to incorporating these findings into TB control in the U.S.

One critical limitation to our study was the availability of more detailed socio-demographic data. While our findings suggest significant social patterning of TB incidence, our ability to infer what may be driving the observed disparities is limited based on the data available. Future studies would benefit from the collection of more detailed socio-demographic data. This could be done by improving the data collected on the RVCT form or research studies with the explicit purpose of collecting such data.

Our incidence rate calculations were based on extrapolations of census data. Updated population denominators were not available for all years, possibly reducing accuracy of the population-level data. However, the resulting bias is likely non-differential, biasing the observed estimates towards the null. Additionally, there are several limitations inherent in the methodology utilized to define a genotypic cluster and thereby proxy recent transmission. Some misclassification of genotypic clustering may have occurred for those cases diagnosed in 2004 and 2012. To be classified as clustered, a case had to share a genotype and a diagnostic date within one year of another case. Thus, there may have been some 2004 cases that were misclassified as non-clustered since we could not link them to cases diagnosed in 2003 because of a lack of data for the 2003 cases. The same would be true of those cases diagnosed in 2012. We performed sensitivity analyses excluding 2004 and 2012 cases. The proportion of cases classified as clustered was not substantially different when excluding 2004 and 2012. Moreover in the multivariable models, the effect estimates were relatively close and were most often an underestimate of the disparity.

Moreover, while we believe that the addition of a time restriction in our genotypic cluster definition increased the specificity of the designation, it is also possible that the use of a one-year time period may systematically underestimate the true proportion of recent transmission. One year is the shortest cut point that has been used to distinguish recent transmission from reactivation disease in previous studies [55, 56]. For example, Borgdorff et al. found a significant portion of secondary cases were diagnosed more than one year after infection from the primary case [57]. In using the one year criterion, we followed a previously published methodology used in the U.S. context so as to allow our analysis to be comparable to previous reports regarding recent transmission trends [7, 30, 58]. We also believe that our use of this classification helps to reduce the effect of the relatively lower discriminatory power of 12-loci MIRU typing on the clustering rate, another methodological limitation that may result in underestimation of recent transmission in our study population.

Future studies should seek to employ multi-faceted methodologies for identifying recent transmission, such as the one described by Yuen et al. [59]. They used a plausible source-case methodology that used data on the genotype, infectiousness status, geographic location and time to classify cases as recent transmission. Accordingly, they found that only 14% of national cases were classified as recent transmission, notably lower than what we, and others, have found.

## Conclusions

This is the first study offering a more in-depth analysis of the trends in TB incidence in Michigan. Our findings suggest that both ongoing transmission and reactivation of LTBI are contributing to the incidence of TB in Michigan. This is consistent with trends in Arkansas where studies have documented that ongoing transmission is occurring as well [7, 30]. In low incidence settings, such as Arkansas and Michigan, TB control is not often focused on reducing transmission of MTB. However, these studies suggest that additional strategies addressing ongoing control of TB transmission should be a key tenet of TB control programs in low incidence, as well as high incidence settings. As the U.S. moves towards TB elimination, more local approaches to TB control are needed that can address the specific socio-demographic composition in a given context and tailor strategies accordingly [28].

Additionally, disparities in incidence persist despite ongoing TB control efforts suggesting more targeted TB control is needed to reduce incidence in high-risk groups. The greater disparities by race and nativity demonstrate some of the ways that TB incidence is socially patterned, disproportionately affecting the most vulnerable members of the population. Reducing these disparities will require a multi-faceted approach encompassing the social and environmental contexts of high-risk populations.

## Additional files

Additional file 1: Table S1. Comparison of the Distribution of Selected Demographic and Clinical Characteristics Among all Reported Cases in Michigan and the Study Sample. (DOCX 15 kb)

Additional file 2: Table S2. Incidence Rate Ratio of TB According to Selected Socio-Demographic Characteristics in Michigan, 2004–2012. Models with and without nativity are shown. (DOCX 16 kb)

## Abbreviations

LTBI: Latent TB infection
SES: Socio-economic status
TB: Tuberculosis
