# Supplementary material for: Race and nativity are major determinants of tuberculosis in the U.S.: evidence of health disparities in tuberculosis incidence in Michigan, 2004–2012
Source: BMC Public Health. 2017 Jun 2;17:538. doi: 10.1186/s12889-017-4461-y (PMC5457589; doi:10.1186/s12889-017-4461-y)
Supplement: Supplementary file 1 — Comparison of the Distribution of Selected Demographic and Clinical Characteristics Among all Reported Cases in Michigan and the Study Sample. (DOCX 15 kb) [file 12889_2017_4461_MOESM1_ESM.docx]

**Supplementary Table 1.** Comparison of the Distribution of Selected Demographic and Clinical Characteristics Among all Reported Cases in Michigan and the Study Sample.

|  | All reported TB cases (n=1,800) | | Study Sample  (n= 1,254) | |
| --- | --- | --- | --- | --- |
| Risk Factor | **No.** | **%** | **No.** | **%** |
| Race |  |  |  |  |
| White | 618 | 34.4 | 419 | 33.4 |
| Black/African American | 721 | 40.1 | 525 | 41.9 |
| Asian | 428 | 23.8 | 310 | 24.7 |
| American Indian/Alaskan Native | 5 | 0.28 |  |  |
| Native Hawaiian or Other | 3 | 0.17 |  |  |
| Unknown | 22 | 1.2 |  |  |
| Missing | 3 |  |  |  |
| Age groups (years) |  |  |  |  |
| <18 | 124 | 6.9 |  |  |
| 18-64 | 1263 | 70.2 | 939 | 74.9 |
| 65+ | 413 | 22.9 | 315 | 25.1 |
| Gender |  |  |  |  |
| Male | 1047 | 58.2 | 750 | 59.8 |
| Female | 752 | 41.8 | 504 | 40.2 |
| Missing | 1 |  |  |  |
| Nativity |  |  |  |  |
| Foreign-born | 798 | 44.5 | 557 | 44.5 |
| US-born | 996 | 55.5 | 694 | 55.5 |
| Missing | 6 |  | 3 |  |
| Site of disease |  |  |  |  |
| Pulmonary | 1188 | 66.2 | 880 | 70.3 |
| Extrapulmonary | 463 | 25.8 | 275 | 22.0 |
| Both | 143 | 8.0 | 97 | 7.8 |
| Missing | 6 |  | 2 |  |
|  |  |  |  |  |
| Classifications of race, age, gender, nativity, and site of TB disease were defined based on Report of Verified Case of TB form developed by the Centers for Disease Control and Prevention. | | | | |
|  | | | | |
