# Supplementary material for: Race and nativity are major determinants of tuberculosis in the U.S.: evidence of health disparities in tuberculosis incidence in Michigan, 2004–2012
Source: BMC Public Health. 2017 Jun 2;17:538. doi: 10.1186/s12889-017-4461-y (PMC5457589; doi:10.1186/s12889-017-4461-y)
Supplement: Supplementary file 2 — Incidence Rate Ratio of TB According to Selected Socio-Demographic Characteristics in Michigan, 2004–2012. Models with and without nativity are shown. (DOCX 16 kb) [file 12889_2017_4461_MOESM2_ESM.docx]

## Supplementary Table 2. Incidence Rate Ratio of TB According to Selected Socio-Demographic Characteristics in Michigan, 2004-2012. Models with and without nativity are shown.

|  |  | | **Clustered Cases** | | **Non-Clustered Cases** | | | |  |
| --- | --- | --- | --- | --- | --- | --- | --- | --- | --- |
|  |  | **Model 1** | | **Model 2** | |  | **Model 1** | **Model 2** | |
| **Variable** | **N** | **IRR (95% CI)** | | **IRR (95% CI)** | | **N** | **IRR (95% CI)** | **IRR (95% CI)** | |
|  | 479 |  | |  | | 775 |  |  | |
| **Race** |  |  | |  | |  |  |  | |
| White | 107 | Ref. | | Ref. | | 312 | Ref. | Ref. | |
| Black | 296 | 37.8 (20.3, 70.4) | | 24.6 (15.5, 38.8) | | 229 | 8.9 (5.7, 14.0) | 7.6 (5.3, 11.1) | |
| Asian | 76 | 11.2 (5.67, 22.1) | | 18.6 (10.5, 32.9) | | 234 | 8.7 (5.4, 14.1) | 23.5 (16.3, 35.8) | |
|  |  |  | |  | |  |  |  | |
| **Nativity** |  |  | |  | |  |  |  | |
| U.S.-born | 359 | - | | Ref. | | 335 | - | Ref. | |
| Foreign-born | 117 | - | | 10.04 (6.6, 15.2) | | 440 | - | 7.0(5.0, 9.8) | |
| *3 missing |  |  | |  | |  |  |  | |
|  |  |  | |  | |  |  |  | |
| **Gender** |  |  | |  | |  |  |  | |
| Male | 313 | Ref. | | Ref. | | 437 | Ref. | Ref. | |
| Female | 166 | 0.60 (0.36, 1.01) | | 0.61 (0.41, 0.91) | | 338 | 0.74 (0.51, 1.1) | 0.74 (0.54, 1.0) | |
|  |  |  | |  | |  |  |  | |
| **Age** |  |  | |  | |  |  |  | |
| 18-64 Years | 414 | Ref. | | Ref. | | 525 | Ref. | Ref. | |
| 65+ Years | 65 | 0.86 (0.48, 1.5) | | 0.83 (0.54, 1.3) | | 250 | 2.8 (1.9, 4.1) | 2.6 (1.9, 3.5) | |
|  |  |  | |  | |  |  |  | |
| Classifications of race, age, gender, nativity, and site of TB disease were defined based on Report of Verified Case of TB form developed by the Centers for Disease Control and Prevention. | | | | | | | | |  |
| Models based on multivariable negative binomial regression models. | | | | | | | | |  |
| Clustered and non-clustered cases were modeled separately with all four demographic characteristics included in models: race, nativity, gender, and age. | | | | | | | | |  |
| IRR = incidence rate ratio | | | | | | | | |  |
| Ref. = reference group | | | | | | | | |  |
